# Comparative long-term oncological outcomes of intraoperative radiotherapy vs. whole-breast irradiation in early breast cancer: a single institute study

**DOI:** 10.3389/fonc.2024.1411598

**Published:** 2024-10-08

**Authors:** Mau-Shin Chi, Hui-Ling Ko, Tsen-Long Yang, Ya-Fang Liu, Kwan-Hwa Chi, Fiona Tsui-Fen Cheng

**Affiliations:** ^1^ Department of Radiation Therapy & Oncology, Shin Kong Wu Ho-Su Memorial Hospital, Taipei, Taiwan; ^2^ Institute of Veterinary Clinical Science, School of Veterinary Medicine, National Taiwan University, Taipei, Taiwan; ^3^ Department of General Surgery, Shin Kong Wu Ho-Su Memorial Hospital, Taipei, Taiwan; ^4^ Department of Research, Shin-Kong Wu Ho-Su Memorial Hospital, Taipei, Taiwan

**Keywords:** breast cancer, ductal carcinoma in situ, radiotherapy, intraoperative radiation therapy, whole breast irradiation

## Abstract

**Background:**

Intraoperative radiation therapy (IORT) and whole breast irradiation (WBI) are both effective adjuvant radiotherapy methods for ductal carcinoma *in situ* (DCIS) or early-stage breast cancer (BC) patients undergoing breast-conserving surgery (BCS). We aim to evaluate the long-term oncological efficacy and refine patient selection criteria based on our findings.

**Methods:**

Female patients who underwent either IORT or WBI from January 2016 to December 2019, with a minimum follow-up of 12 months were collected. IORT was administered as a single fraction of 20 Gray (Gy) to the lumpectomy cavity using the Axxent electronic brachytherapy system, while WBI consisted of a standard fractionation of 50 Gy in 25 fractions, along with a reduced boost of 10 Gy. The clinicopathologic characteristics and oncological outcomes were retrospectively analyzed.

**Results:**

A total of 247 patients were enrolled, comprising 164 with BC and 83 with DCIS. Among them, 112 underwent IORT, and 135 received WBI after BCS. The median age was 62.2 years, with median tumor sizes of 1.5 cm for BC and 1.2 cm for DCIS. At a median follow-up of 64.6 months, IORT demonstrated 11 locoregional recurrences (LRR), 1 metastasis, and 1 death, compared to 4 LRR, 5 metastases, and 2 deaths in the WBI group. WBI yielded significantly higher locoregional control (97.0% vs. 90.2%, p = 0.033), although metastasis-free (96.3% vs. 99.1%, p = 0.166) and overall survival rates (98.4% vs. 99%, p = 0.688) did not differ. The LRR rate was significantly higher in the IORT group among the DCIS or BC patients (p = 0.043). The hazard ratio for locoregional recurrence significantly increased in estrogen-receptor-negative (ER-) patients in both univariate analysis (HR = 4.98, 95% CI = 1.76-14.09, p = 0.002) and multivariate analysis (HR = 40.88, 95% CI = 1.29-1297.84, p = 0.035). Additionally, IORT was associated with increased LRR in the multivariate analysis (HR = 4.71, 95% CI = 1.16-19.06, p = 0.030).

**Conclusion:**

At a long-term follow-up, the LRR rate was higher in the BCS followed by IORT, without significant differences in metastasis-free or overall survival rates. Our data confirmed the importance of exclusion ER- patients for IORT.

## Introduction

Treatment for early-stage breast cancer (BC) or ductal carcinoma *in situ* (DCIS) involving breast conservation surgery (BCS) followed with adjuvant radiotherapy (RT) or modified radical mastectomy (1, 2). Adjuvant RT halve the 10-year disease recurrence rate and decrease the 15-year BC death rates (3). Published literatures even suggests a survival benefit of breast conserving therapy over mastectomy in early-stage patients (4, 5). However, a conventional 4- to 6-week RT regimen can be challenging for some patients, leading to the exploration of accelerated irradiation options, such as ultra-hypofractionation (26-28.5 Gy in 5 fractions) or intraoperative radiation therapy (IORT) (6–10). A 1-week ultra-hypofractionation course is non-inferior to the standard 3-week schedule in terms of 5-year tumor recurrence (9, 10). Nonetheless, the single-session treatment by IORT may offer a more convenient and less disruptive alternative to whole breast irradiation (WBI).

IORT delivered either by electron or photon beams, administers a singular high-dose radiation to a reduced breast volume during BCS. Rigorous accelerated partial breast irradiation (APBI) criteria, endorsed by the American Society for Radiation Oncology (ASTRO) and the European Society for Radiotherapy & Oncology (ESTRO), support its application in highly selected early-stage BC (11–13). IORT has demonstrated favorable cosmetic outcomes, likely due to the small treatment volumes and complete skin sparing (12). A meta-analysis by Zhang et al. emphasized fewer side effects, better cosmetic outcomes, and similar mortality rates with IORT compared to whole breast irradiation (WBI) (14). The long-term findings from two pivotal trials, the TARGIT-A (photon IORT) and ELIOT (electron IORT), have been published. The TARGIT-A trial showed similar outcomes between IORT and (WBI) (15, 16), while the ELIOT trial found a higher 10-year local recurrence rate of 8.1% in the IORT group compared to 1.1% in the WBI group (17). Extended follow-up in other reports generally reported a higher locoregional recurrence (LRR) rate with IORT (18–21). Considering increased patient convenience and preference, even with the potential for a higher LRR risk, it may be reasonable to conduct IORT in a prospective clinical trial or multi-institutional registry (13).

Based upon our prior data, we have demonstrated a 1.9% recurrence rate by IORT with Axxent electronic brachytherapy system (Xoft/iCad, Inc., San Jose, CA) in a 31.1-month follow-up (22). This article aims to contribute a long-term follow-up of the oncological outcome in comparison with WBI, and optimization the patient selection criteria for IORT.

## Patients and methods

### Patient selection

From January 2016 to January 2020, patients with stage I, II BC or DCIS who underwent BCS followed by either IORT or WBI were retrospectively reviewed. Collected data included the patient’s clinical pathological status and oncological outcomes within a minimum follow-up of 12 months. Each patient is required for preoperative mammography and breast sonography. The institute adjusted its IORT inclusion criteria based on the suitable and cautionary groups outlined in the ASTRO consensus guideline for APBI (11). These require patients older than 45 years old, with unifocal DCIS or BC less than 4 cm, without lymph node involvement on preoperative images, and a negative sentinel lymph node biopsy (SLNB). Criteria for WBI in this study required DCIS or BC less than 4 cm, with RT targeting only the breast. Although all the IORT cases qualified for standard WBI, potential candidates were informed of both treatment modalities by the surgeon or radiation oncologist prior to surgery, with the understanding that IORT would not be administered if SLNB was positive. This study was approved by the Institutional Review Board.

### Surgical treatment

BCS was carried down to the level of the pectoralis fascia, and an intraoperative frozen section for margin status was done for both DCIS and BC. SLNB with frozen-section diagnoses was done for BC. For IORT patients, only a negative SLNB would proceed to treatment. Per institutional protocol, a negative microscopic margin was highly recommended, and re-excision was strongly suggested for positive margins.

### Radiotherapy

IORT was carried out by Axxent electronic brachytherapy system. The Axxent system uses a miniature X-ray source of 50kVp to deliver a single dose of 20 grays (Gy) by a balloon applicator. A balloon-to-tissue apposition was done by retention sutures to maintain a minimum 10mm balloon-to-skin distance. A flexible lead shield was placed for radioprotection. Before beam-on, the balloon will be checked for its reproducibility. The lead shield, applicator balloon, and retention sutures were removed after IORT.

WBI was administered using either the Elekta Synergy (Elekta, Stockholm, Sweden) with image-guided, volumetric modulated arc therapy or TomoTherapy (Accuray, Sunnyvale, CA, USA), both employing standard immobilization devices. Target volumes were defined according to the Radiation Therapy Oncology Group contouring atlas (23). For patients with left-sided DCIS or BC, a deep-inspiration breath-hold technique was recommended for cardiac protection. By institute’s protocol, all patients received standard fractionation of 50 Gy in 25 fractions with a 10 Gy boost to the primary tumor bed. The regional node was not irradiated due to node negativity.

### Adjuvant treatment and follow-up

Postoperatively, all patients with positive hormone receptors received endocrine therapy. Adjuvant chemotherapy was recommended for BC patients with tumors larger than 1 cm in diameter or positive nodal metastases on final pathology. Adjuvant trastuzumab was suggested for positive HER-2 BC patients according to guidelines. Oncotype Dx stratification was not performed at the time of analysis. If nodal metastases were identified in the final pathology, an axillary nodal dissection was performed. Adjuvant WBI and regional nodal irradiation were mandatory for patients with four or more positive nodes and strongly recommended for those with one to three positive nodes following systemic treatment. After treatment, patients attend regular clinical check-ups every 3 months, breast sonography every 3 to 6 months, and yearly mammography. For recurrent or metastatic disease, the standard salvage treatments were prescribed.

### Endpoints

The primary endpoint was the LRR rate between the two treatment groups. Recurrence in the lumpectomy site was defined as a true local recurrence, recurrences within the ipsilateral breast and the lumpectomy field was defined as secondary local recurrence, and ipsilateral regional nodal recurrences were defined as regional recurrence. The secondary endpoints include metastatic-free survival, overall survival, and treatment-related side effects.

### Statistical analysis

The Statistical analysis was conducted using SPSS for Windows version 26.0 (SPSS Inc., Chicago, IL) software. The continuous variables of the clinical-pathological status were compared by t-test and Mann-Whitney U test. The categorical variables were compared by the Chi-squared test or Fisher’s exact test. Kaplan-Meier analysis was used to measure the cumulative risks of recurrences and survival between two groups and the log-rank test was used to examine the two curves. A Cox proportional hazard model was used to investigate the associations between the two groups. A p-value < 0.05 was considered statistically significant.

## Results

### Patient characteristics

A total of 249 patients were included in the study, with 166 diagnosed with BC and 83 with DCIS. Among the 114 patients in the IORT group, 2 were lost to follow-up, while the WBI group comprised 135 patients, resulting in a final analyzed patient count of 247. The demographics and clinical characteristics are detailed in **Table 1**. The median age was 62.2 years (range: 46–81 years), with median tumor sizes of 1.5 cm for IDC and 1.2 cm for DCIS. Ki-67 levels and lymphovascular invasion data were not mandatory on the pathology report before 2019, leading to insufficient data for univariate and multivariate analysis. As this is a retrospective study, some patients fell outside the ASTRO-defined “suitable” category for IORT. Of the IORT group, 17 patients exhibited a single positive nodal involvement on final pathology, categorized as N0 i^+^ (1 patient), N1mi (3 patients), and N1a (13 patients).

**Table 1:** Patient demographics and clinical characteristics (N=247).

Variables (mean ± SD, n(%))	IORT (n=112)	WBI (n=135)	p value
Age	63.5 ± 9.1	61.6 ± 10.1	0.207
<=49	3(2.7)	15(11.1)	
50-59	41(36.6)	49(36.3)	
>=60	68(60.7)	71(52.6)	
Cancer Type			0.225
DCIS	33(29.5)	50(37.0)	
IDC	79(70.5)	85(63.0)	
Tumor size (cm)			0.320
<0.5	11(9.8)	11(8.1)	
0.5–1	19(16.9)	17(12.6)	
>1-2	57(50.8)	66(48.9)	
>2	25(22.5)	41(30.4)	
DCIS tumor size (cm)	1.1	1.25	0.473
<0.5	7(21.2)	9(18.0)	
0.5–1	5(15.2)	6(12.0)	
>1-2	16(48.5)	20(40.0)	
>2	5(15.2)	15(30.0)	
IDC tumor size (cm)	1.5	1.5	0.595
<0.5	4(5.1)	2(2.4)	
0.5–1	14(17.7)	11(12.9)	
>1-2	41(51.9)	46(54.1)	
>2	20(25.3)	26(30.6)	
pT stage			0.229
Tis	33(29.5)	50(37.0)	
T1a	2(1.8)	4(3.0)	
T1b	22(19.6)	17(12.6)	
T1c	41(36.6)	41(30.4)	
T2	13(11.5)	23(17.0)	
Grade			0.003
1	24(21.4)	23(17.0)	
2	72(64.3)	65(48.1)	
3	12(10.7)	35(25.9)	
NA	4(3.6)	12(8.9)	
Resection margin			0.035
Positive	8(7.1)	22(16.3)	
Negative	104(92.9)	110(81.5)	
NA	0	3(2.0)	
ER status			0.086
Positive	99(88.3)	106(78.5)	
Negative	13(11.7)	27(20)	
NA	0	2(1.5)	
PR status			0.009
Positive	94(83.9)	92(68.1)	
Negative	18(16.1)	41(30.4)	
NA	0	2(1.5)	
Her-2 status			<0.001
Positive	24(21.4)	64(47.4)	
Negative	88(78.6)	71(52.6)	
NA	0	0	
Lymphovascular invasion			0.285
Positive	16(14.3)	3(2.2)	
Negative	93(83.0)	39(28.9)	
NA	3 (2.7)	93(68.9)	
Ki67 index			0.065
<14	51(45.5)	37(27.4)	
>14	32(28.6)	44(32.6)	
NA	29(25.9)	54(40)	
ASTRO APBI Risk Group			0.003
Suitable	69(61.6)	54(40)	
Cautionary	19(17.0)	40(29.6)	
Unsuitable	24(21.4)	41(30.4)	
Median Follow-up time(months)	65.1 ± 17.1	63.6 ± 16.3	0.481

### Oncological outcomes

**Figure 1:**
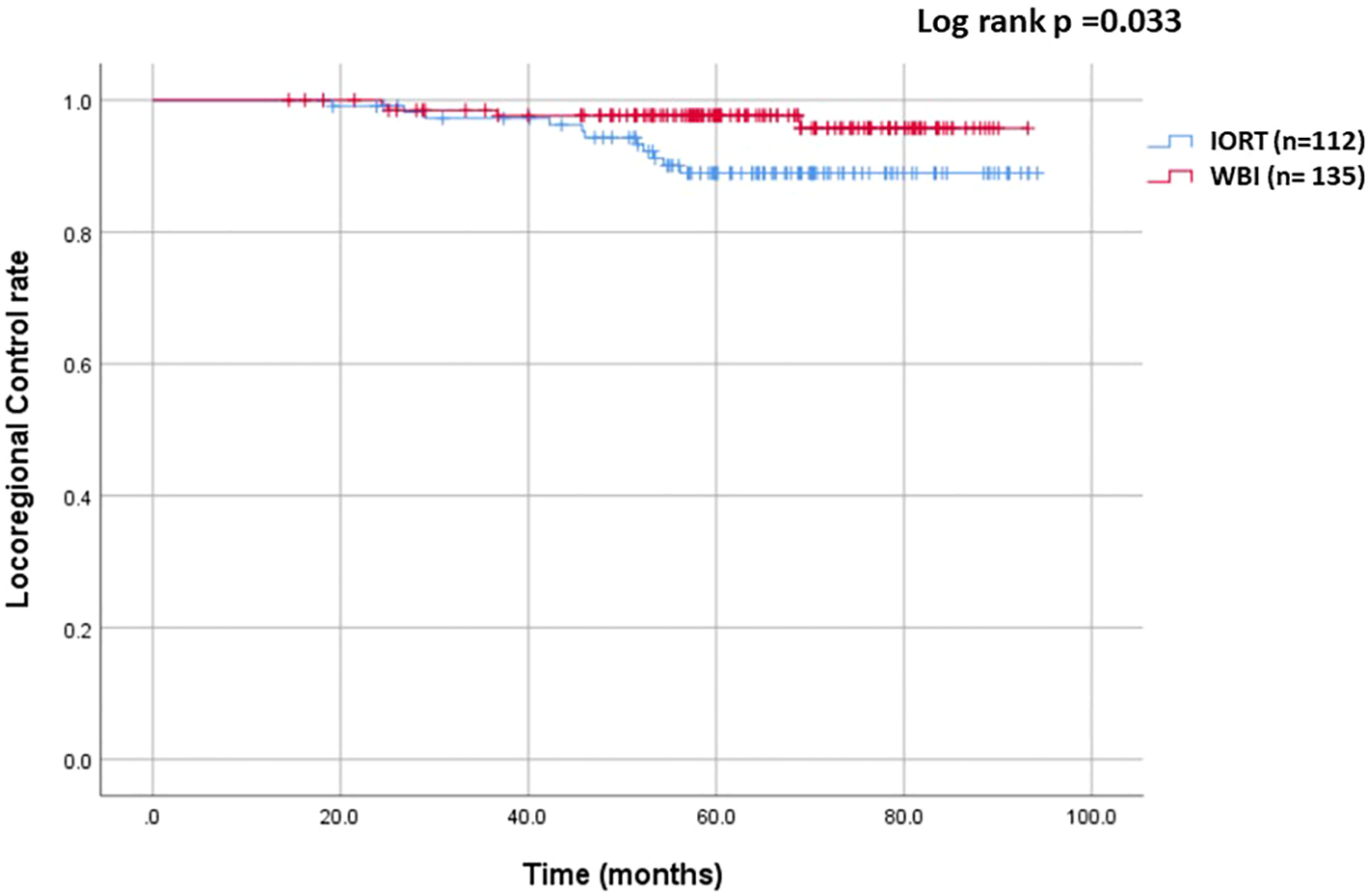
Cumulative incidence of locoregional control in the IORT and WBI groups. The locoregional control rate was significantly higher with WBI than with IORT (97.0% vs. 90.2%, p = 0.033).

**Figure 2:**
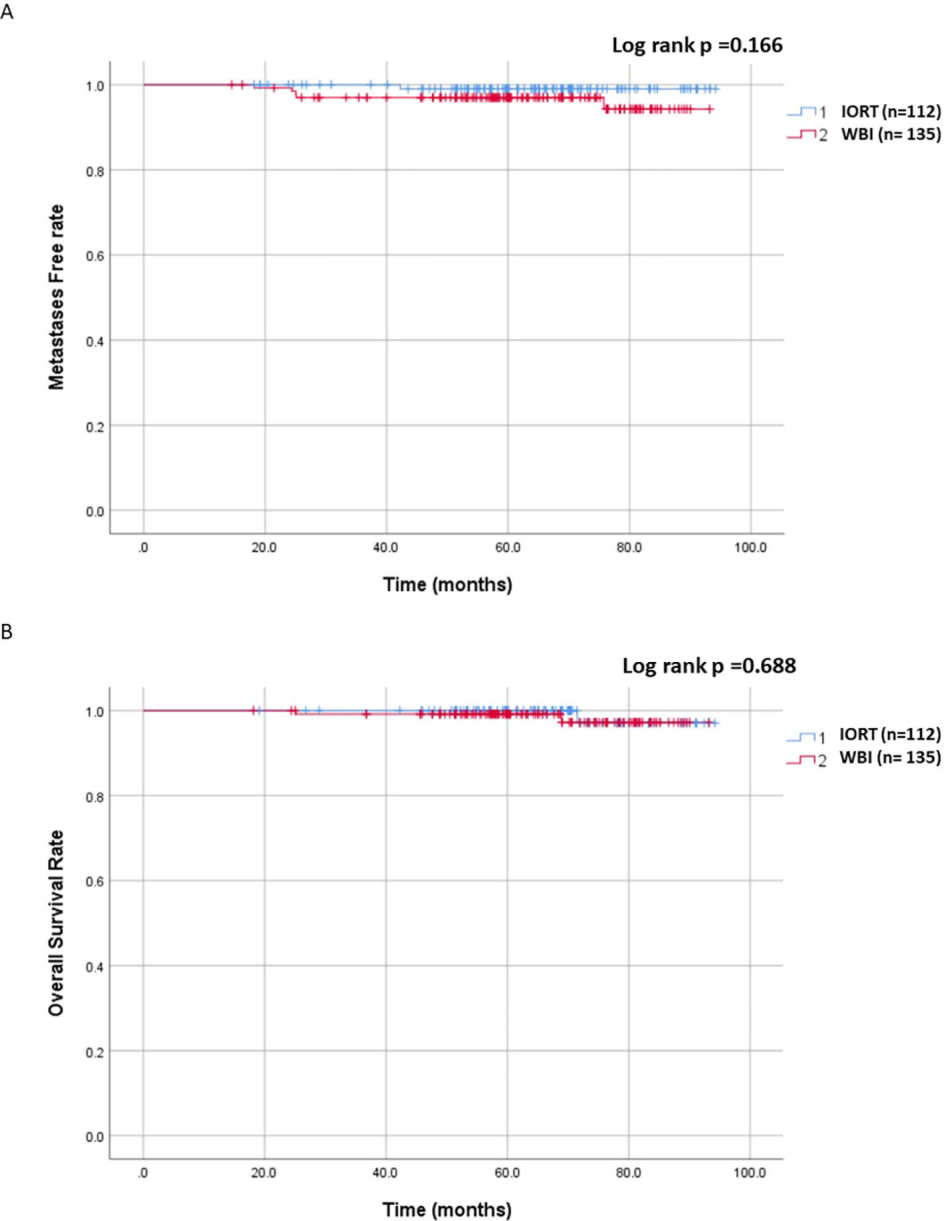
Cumulative incidence of metastasis-free and overall survival in the IORT and WBI groups. **(A)** No significant differences were observed in metastasis-free survival between WBI and IORT (96.3% vs. 99.1%, p = 0.166). **(B)** No significant differences were observed in overall survival rates between WBI and IORT (98.4% vs. 99%, p = 0.688).

The median follow-up was 64.6 months (range: 13.6 - 94.2 months, mean of 64.3 months). In the IORT group, there were 11 LRR, 1 metastasis, and 1 death, compared to 4 LRR, 5 metastases, and 2 deaths in the WBI group. Locoregional control rate was significantly higher in the WBI group (97.0% vs. 90.2%, p = 0.033), as depicted in **Figure 1**. **Figure 2** demonstrated no significant differences in metastasis-free survival (96.3% vs. 99.1%, p = 0.166) or overall survival rates (98.4% vs. 99%, p = 0.688) between WBI and IORT, respectively.

Stratified analysis showed a notably higher LRR in the IORT group among DCIS and BC patients (p= 0.043). Locoregional control rates were 95.2% (80 out of 83) for DCIS patients, with 93.9% (31 out of 33) in IORT and 98% (49 out of 50) in WBI. Among all BC patients, the locoregional control rate stood at 92.7% (152 out of 164), with 88.6% (70 out of 79) in the IORT group and 96.5% (82 out of 85) in the WBI group.

When stratifying LRR into local or regional recurrences, there were no significant differences between IORT and WBI for local recurrence (p = 0.235). However, the regional recurrence rate was statistically higher with IORT (p = 0.03). In the IORT group, one DCIS and five BC patients experienced true local recurrences, and one DCIS patient had a secondary local recurrence; all were successfully salvaged with surgery. Among the 17 patients with single nodal metastases, all received adjuvant chemotherapy, resulting in only one local recurrence, which was successfully treated with surgery. Two patients also received adjuvant radiotherapy after chemotherapy; one received radiation to the breast and regional nodes, while the other received only regional nodal irradiation. In the WBI group, true local recurrence occurred in 1 DCIS case and 3 BC cases and were managed surgically.

A statistically significant difference was observed in the locoregional control rate when stratified by the ASTRO APBI risk group (p= 0.038). Within the suitable subgroup, the control rate was 95.1% (6 recurrences out of 123 patients), with 91.3% (6/69) in the IORT group and 100% (0/54) in the WBI group. For the cautionary subgroup, the control rate stood at 89.8% (6/59), with 78.9% (4/19) in the IORT group and 95% (2/40) in the WBI group. In the unsuitable subgroup, the control rate reached 95.3% (3/65), with 95.8% (1/24) in the IORT group and 95.1% (2/41) in the WBI group.

In the IORT group, one local recurrent patient developed lung metastases one year later after salvage surgery and WBI. The metastatic lesion was surgically salvaged, and she is currently undergoing chemotherapy. The single mortality case in the IORT group was not related to BC. In the WBI group, there were four cases of metastasis, in which 2 were from the 3 cases with local recurrence. The remaining two cases had no LRR, which resulted in one cancer-related death. The other fatality was not related to BC.

### Risk factors of locoregional recurrence

In the univariate analysis, estrogen-receptor negative (ER-) patients showed a significantly elevated hazard ratio for LRR (HR = 4.98, 95% CI=1.76 -14.09, p = 0.002). In the multivariate analysis, IORT (HR = 4.71, 95% CI = 1.16 -19.06, p = 0.030) and ER- (HR = 40.88, 95% CI = 1.29 -1297.84, p = 0.035) were associated with increased LRR. There were no statistical differences based on age, tumor size, margins, HER-2 subtype, or ASTRO APBI risk groups in both univariate and multivariate analyses for both IORT and WBI, as shown in **Table 2**.

**Table 2:** Univariate and Multivariate analyses of risk factors for local recurrence.

	Crude HR (95 CI)	p value	Adjusted HR (95 CI)	p value
RT Modality				
WBI	Reference			
IORT	2.70(0.85, 8.54)	0.091	4.71(1.16, 19.06)	0.030
Tumor size (mm)	1.26(0.77, 2.04)	0.359	1.26(0.627, 2.51)	0.521
Resection margin				
Positive	Reference			
Negative	0.55	0.364	0.05(0.002, 1.03)	0.052
ER status				
Positive	Reference			
Negative	4.98(1.76, 14.09)	0.002	40.88(1.29, 1297.84)	0.035
PR status				
Positive	Reference			
Negative	2.38(0.85, 6.69)	0.101	0.12(0.004, 3.90)	0.235
Her-2 status				
Positive	Reference			
Negative	1.56(0.49, 4.92)	0.453	0.94(0.25, 3.55)	0.930
ASTRO APBI Risk Group				
Unsuitable	Reference			
Suitable	1.13(0.28, 4.55)	0.859	6.09(0.26, 140.62)	0.169
Cautionary	1.62(0.401, 6.52)	0.499	9.30(0.46, 188.04)	0.146

### Side Effects

After treatment, both groups experienced no side effects beyond grade 2 dermatitis. Throughout the follow-up period, there were no occurrences of rib fractures, wound infections, or fat necrosis. Among the two patients in the IORT group who underwent salvage RT for LRR, a grade 1 dermatitis and grade 1 esophagitis were documented during treatment and resolved within one-month post-RT.

## Discussion

This study represents the largest comparison between IORT and WBI conducted at a single institute in Taiwan. Our prior findings showed a 1.9% rate of locoregional recurrence (LRR) for IORT, with a mean follow-up duration of 31.1 months (22). Subsequent observations during extended follow-up revealed an increased LRR rate of 9.8% over a mean follow-up period of 64.3 months, providing long-term insights into oncological outcomes.

The treatment landscape for early-stage BC emphasizes minimizing treatment intensity, making IORT an appealing option (7, 24, 25). Its popularity surged tenfold in the USA from 2010 to 2013, highlighting its attraction for a shorter treatment course (25). IORT offers advantages in avoiding geographical and temporal misses, along with a radiobiological advantage of single high-dose treatment, while reducing radiation exposure to the internal organs (26, 27). It ensures timely RT completion while reducing risks of viral exposure, which is crucial during the COVID pandemic (28). Economically, IORT enhance patients’ quality of life while conserving healthcare resources (29, 30). Notably, the Taiwan IORT Study Cooperative Group pointed out patients’ preference toward IORT are often due to work-related considerations (31). This was also evident form our analysis, with 44.9% of all treated early BC patients (62 out of 138) choosing IORT from the period of January 2018 to December 2019.

An additive benefit of IORT for local control could be expected. Preclinical studies have shown that IORT influences wound response by downregulating miR-223, which in turn reduces the activation of the epidermal growth factor receptor and disrupts the tumor growth-stimulating loop (32). Furthermore, analysis of surgical fluids from IORT-treated patients has indicated inhibition of breast cancer cell growth and motility through alterations in cytokine expression and intracellular signaling pathways (33).

Under a stringent institutional selection criterion, the majority IORT participants were over 60 years old, had tumors less than 2cm in size, had negative margins, and belonged to the ASTRO suitable risk profile. In line with the published data (17–19), although the overall survival and metastasis-free rate were both high and comparable, the LRR was higher after IORT. Meta-analysis also reported a higher recurrence rate over a median follow-up of 8.6 years in the IORT group (34), concluding that IORT is suitable for the selected low-risk BCs, consistent with the ASTRO and ESTRO guidelines (11, 12). The updated TARGIT-A and ELIOT trials similarly highlight an increased LRR rate following IORT in patients within the ASTRO unsuitable or cautionary risk group (16, 17). A study by Daphne et al. comparing APBI with external beam radiation to IORT reported a local recurrence rate of 10.6% in the IORT group compared to 3.7% with external beam at 5 years, with no significant differences in distant recurrence or overall survival (35). Consistent with our findings, these studies highlight the critical role of patient selection and treatment protocols in shaping outcomes.

The significance of excluding ER- patients for IORT was supported by statistically significant risk of LRR in the *post hoc* analysis. Patients with a negative hormone receptor have an increased risk of recurrence within the first 5 years (36). In a study by Cannon et al., which included 277 breast cancer cases treated with APBI using high dose rate brachytherapy, an ER- status was strongly linked to LRR in the multivariate analysis (37). Likewise, another study involving 147 patients treated with IORT found correlations between negative hormone receptors, axillary node involvement, positive margins, and lymphovascular invasion with LRR (38).

While the local control rates were similar, the IORT group experienced significantly more regional recurrences. Nodal metastases was a notable risk factor for LRR (39). Despite the institute’s protocol mandating a negative SLNB for IORT eligibility, 15.2% of patients exhibited a single nodal metastasis upon the final pathology, surpassing the rates reported in other studies (31, 40–45). Although the TARGIT-A and ELIOT trials indicated that lymph node involvement of less than three does not necessary contraindicate IORT, patients with positive nodes typically require more aggressive treatment (40, 41, 46). Consistent with this, among the 17 patients with positive nodal involvement in the analysis, all underwent salvage surgery and chemotherapy, with two also received irradiation, resulting in only one case of local recurrence.

Given the unknown final histopathology at the time of IORT, a risk adapted IORT protocol may be crucial for the high-risk patients. Considering a higher LRR associated with IORT, the 2024 ASTRO APBI guideline does not recommend kV IORT alone (without WBI) or electron IORT for early-stage BC receiving PBI, unless part of a clinical trial or multi-institutional registry (13). While awaiting the results of the TARGIT-B trial (NCT01792726), the European group of the International Society of Intraoperative Radiation Therapy has integrated IORT as tumor bed boost administered before WBI, particularly in Grade 3 tumor or triple-negative BCs. The study demonstrated excellent long-term tumor control rates of 95% (12). Silverstein et al. had reported on a series of 1600 cases treated with IORT, of which 207 high-risk patients received additional WBI. This resulted in only two local recurrences, with a 5-year LRR rate of 0.5% (47). In another study, Stoian et al. compared IORT at 20 Gy followed by WBI or simultaneous integrated boost with WBI (SIBRT) in high-risk patients, also reported comparable 5-year local control rates (93% with IORT, 98% with SIBRT) (48). Additionally, for patients with DCIS who underwent IORT and had positive margins, both salvage mastectomy and adjuvant WBI achieved a favorable 3-year local control rate of 94.3% (49).

While our experience with IORT yielded an acceptable locoregional control rate during a 5-year follow-up period, several limitations warrant consideration. Firstly, despite employing stringent criteria for patient selection in our retrospective data collection, a selection bias exists. Secondly, the analysis was confined to a single institution, and the WBI regimen administered followed only by the conventional fractionation. Thirdly, the substantial amount of missing data regarding ki67 levels and lymphovascular invasion in the WBI group necessitated their exclusion from the *post hoc* analysis. Lastly, despite requiring a negative SLNB for IORT, seventeen patients were found to have single-node involvement on final pathology, placing them in the unsuitable risk group. In this group, 15 patients did not receive additional RT. Although the LRR at the follow-up time was comparable between IORT group (95.8%) and WBI group (95.1%), a longer follow-up is needed to fully assess outcomes.

In conclusion, while the rate of LRR is higher with IORT compared to WBI, the rates of metastasis and mortality are similar. IORT could be considered a viable alternative to WBI, particularly with careful patient selection, thorough counseling, and the exclusion of ER- patients. Additionally, IORT may serve as a beneficial tumor bed boost option for the high-risk patients.

## Data Availability

The raw data supporting the conclusions of this article will be made available by the authors, without undue reservation.
